# Synthesis and Antibacterial Activity of Metal-Containing Ultraviolet-Cured Wood Floor Coatings

**DOI:** 10.3390/polym13183022

**Published:** 2021-09-07

**Authors:** Chia-Wei Chang, Kun-Tsung Lu

**Affiliations:** Department of Forestry, National Chung Hsing University, 250 Kuo-Kuang Rd., Taichung 402, Taiwan; dimmerc@hotmail.com

**Keywords:** zinc mono(hydroxyethoxyethyl)phthalate [Zn(HEEP)_2_], antibacterial activity, UV wood floor coating, film properties

## Abstract

In our previous report, the antibacterial agents with different metals, mono(hydroxyethoxyethyl)phthalate [M(HEEP)_2_, M = Zn, Mn, and Ca], were synthesized. For increasing their yields, modified synthesis and purified processes were further investigated. The result of energy-dispersive X-ray spectroscopy showed the M(HEEP)_2_ could be stable and successfully synthesized, and their yields were raised to 73–85% from our previous report of 43–55%. For ultraviolet-cured wood floor coating application, the Zn(HEEP)_2_ was selected as an antibacterial agent and mixed with commercial UV wood floor coating. The effects on the antibacterial activity of UV films with different Zn(HEEP)_2_ additions of 0, 4, 8, and 12 phr as well as the commercial nano-Ag of 12 phr against *Escherichia coli* were evaluated. In the static antibacterial test, the UV films with Zn(HEEP)_2_ additions had similar antibacterial activity of 57–59%. In another dynamic shaking antibacterial test, the film containing 12 phr Zn(HEEP)_2_ had the best antibacterial activity among all the UV films. On the film properties, the Zn(HEEP)_2_-containing UV films had lower gloss and abrasion resistance, and slightly increased the hardness than those of UV film without Zn(HEEP)_2_ addition. However, there were no noticeable differences in mass retention, lightfastness, and thermal stability between UV films with and without the Zn(HEEP)_2_ addition. In this study, the 12 phr Zn(HEEP)_2_-containing UV film provided the best antibacterial activity against *E. coli* and had the balanced film properties for application on the UV wood floor coating.

## 1. Introduction

The addition of antibacterial agents to the resin-based composites has been widely concerned in the medical and dentistry fields [1]. The reports [2,3,4] suggest that the developments of antibacterial agents are composed of organic polymer without compromising their mechanical or physical properties and have a long-term effect on antibacterial efficiency. In the food-packing industry, texture, and functional coating fields, introducing antimicrobial function is expected to decrease the growth of bacteria and fungi [5,6,7,8]. The goal of these applications is to achieve a balance between the antibacterial ability and mechanical properties. The antibacterial agents applied on the wood preservation and finishing are derived from natural components and inorganic metals, which have been widely studied. The natural components of the antibacterial agents have the advantages of being eco-friendly, biodegradable, and posing a low risk to human health [9,10]. However, these natural antibacterial agents, such as chitosan, sorbitol, and essential oils, etc., have poor storability, thermal resistance, and easily cause the color change of substrate. On the contrary, the inorganic antibacterial agents, which are combined with carriers by physical adsorption or ion exchange of Ag, Cu, and Zn metals, have many benefits, including high antibacterial activity, thermal resistance, storability, and versatility without causing antibiotic resistance [5,11,12,13,14]. Consequently, their developments, especially in the nano-Ag particles, move forward rapidly.

In recent years, the fast development of nanotechnology promotes the wide application of nano-metal in antibacterial applications. The nano-metal has excellent antibacterial activity due to the multiple ways of affecting cells, including interrupting the DNA replication through transmitting in the cell, acting as a catalyst, generating reactive oxygen species to damage the cell membrane, and changing the membrane permeability of microorganisms [15,16,17]. Until now, the most representative antibacterial metal was nano-Ag, which is highly toxic, and the research [18] exhibits that the nano-Ag significantly depletes glutathione levels, reduces mitochondrial membrane potential, and increases in reactive oxygen species levels in liver cells of mice. Some researchers [19,20] indicate that the nano-Ag causes hepatotoxicity and passes through the blood-brain barrier of the human body. In addition, the water emission of nano-Ag damages the alga, embryo of fish, crustaceans [21,22,23], and changes the composition of microbes in the soil [24]. Therefore, the concentration of nano-Ag under exposure on humans has been restricted by more and more limitations of law [25]. Although the defects of nano-Ag have been discussed, these factors never delay the development of nano-Ag due to its excellent antibacterial efficiency. However, its expensive cost and the compatibility with the coating resins limits its application on the wood finishing industry.

In our previous report [26], the antibacterial agents, mono(hydroxyethoxyethyl)phthalate [M(HEEP)_2_, M = Zn, Mn, Pb, and Ca], were synthesized, while their yields were only 43–55%. In this study, for increasing the yields of M(HEEP)_2_, an additional synthesized equipment and modified method by using water-free organic eluents, such as ethyl acetate, to replace the acetone and methanol used in our previous study at the purification process were examined. In addition, ultraviolet-cured coatings (UV coatings) which are energy-saving, high production, fast curing, and high quality are very suitable for wood floor finishing. Therefore, the Zn(HEEP)_2_ was selected and combined with the commercial UV coating as a functional UV wood-floor coating. The antibacterial activity of the UV films with the different Zn(HEEP)_2_ additions against *Escherichia coli* and the comparison with adding commercial nano-Ag as a control group were evaluated. Furthermore, the mechanical properties, lightfastness, and thermal stability of the UV films with different Zn(HEEP)_2_ additions were also examined.

## 2. Materials and Methods

### 2.1. Materials

The photoinitiator-containing UV coating with the major component of acrylate resin was provided from UA Wood Floors, Inc. (Yunlin, Taiwan). Diethylene glycol (DEG) and the hydrated divalent metal salts including calcium acetate, manganese acetate, and zinc acetate were obtained from Choneye Pure Chemicals Co. Ltd. (Taichung, Taiwan). Phthalic anhydride (PA) was purchased from Shimakyu’s Pure Chemicals Co. (Osaka, Japan). The commercial nano-Ag composited with zirconium phosphate, which contained 6% Ag, was supplied by Chin-Tai Resins Chemical Co. Ltd. (Taichung, Taiwan). Acetone, ethyl acetate, and xylene were purchased from Union Chemical Works Ltd. (Taichung, Taiwan). Lysogeny brot (LB) was obtained from Merck Taiwan Ltd. (Taipei, Taiwan). *E. coli* (Gram-negative bacterium, ATCC25922) was obtained from the Food Industry Research and Development Institute (Hsinchu, Taiwan). All of the specimen substrates, including glass sheets (Ming Tai Glass Co., Taichung, Taiwan, 15 cm × 10 cm × 0.2 cm), S-16 wear-resistant steel plates (Jiin Liang Industrial Inc., Taipei, Taiwan), polyethylene terephthalate (PET) plates, and Teflon sheets (15 cm × 15 cm × 0.1 cm) (Sheng Huei Instrument Corp., Taichung, Taiwan) were prepared as the experimental substrates, as specified by the CNS 9007 Standard [27]. 

### 2.2. Manufacture of Mono(hydroxyethoxyethyl)phthalate [M(HEEP)_2_]

**Figure 1:**
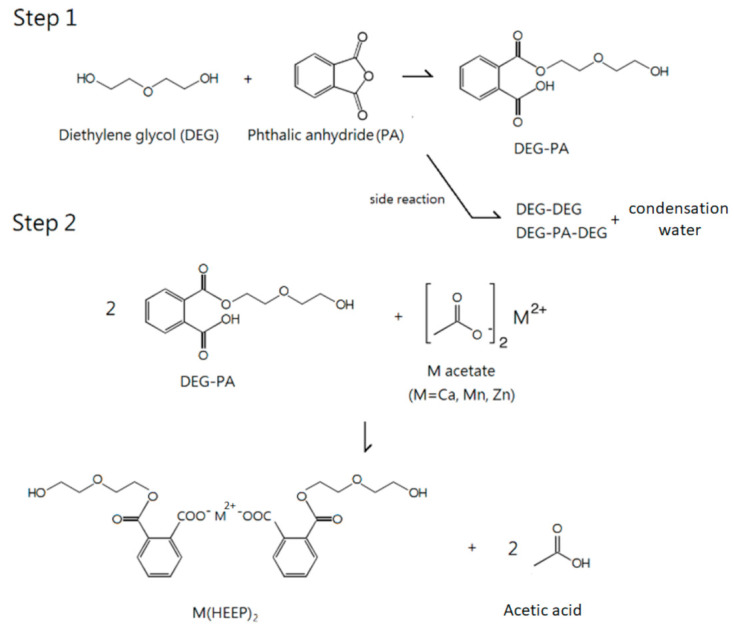
The synthesized process of M(HEEP)_2_.

The 4 moles of DEG and 1 mole of PA were first mixed in a four-neck reaction flask (Sheng Huei Instrument Corp., Taichung, Taiwan) and heated to a constant temperature of 135 °C for 1.5 h. Secondly, 0.5 moles divalent metal acetates including calcium acetate, manganese acetate, and zinc acetate were added, respectively. Then the reaction temperature was raised to 162 °C for calcium acetate, 150 °C for zinc acetate, and 140 °C for manganese acetate, respectively, and all the reactions were maintained for 3 h. During this process, modified equipment by introducing a glass water separator (Sheng Huei Instrument Corp., Taichung, Taiwan) and using thermal insulation materials to maintain the whole system temperature at 120 °C was used for collecting the condensation water completely. Finally, the ethyl acetate and xylene were used to wash the cooled mixture repeatedly. The M(HEEP)_2_ powders were dried in a 60 °C oven (Sheng Huei Instrument Corp., Taichung, Taiwan) for 3 days. The synthesized process is listed in Figure 1.

### 2.3. Identification of M(HEEP)_2_

The M(HEEP)_2_ were identified by using energy-dispersive X-ray spectroscopy (EDS, Oxford X-Act 10 mm^2^, Oxford Instruments, Oxfordshire, England). The field emission scanning electron microscopes (FE-SEM) analysis was carried out by an electron microscope SM-200 (JEOL JSM-6700F, Japan Electron Optics Laboratory Co., Ltd., Tokyo, Japan).

### 2.4. Formulation of the UV Coatings

The Zn(HEEP)_2_ was chosen to evaluate the antibacterial activity against *E. coli* and film properties for the UV wood floor coating application. The Zn(HEEP)_2_ powders were first dissolved in the deionized water with 50 wt.%. Then, the Zn(HEEP)_2_ solutions were added into the commercial UV coating with the addition of 0, 4, 8, and 12 phr, respectively, which were stirred under 600 rpm for 5 min. The UV coating with 12 phr commercial nano-Ag was also prepared as a control group. The substrates were finished by a universal applicator (B-3530, Elcometer, Manchester, UK) with a wet thickness of 100 μm. The specimens were first oven-dried at 50 °C for 10 min and then cured by UV equipment (UVC-362W, C-Sun Mfg., Ltd., Taichung, Taiwan) with a medium-pressure mercury lamp (0.41 J/cm^2^). The radiation distance was 10 cm, and the conveyor speed was 8 m/min, which corresponded to 6 sec irradiation for each specimen. All of the cured specimens were conditioned at a constant temperature of 25 °C for 7 days.

### 2.5. Antibacterial Activity Determination of UV Films

#### 2.5.1. Preparation of Bacterial Strain

The *E. coli* from the frozen culture medium was transferred to LB agar and incubated at 37 °C for 18 h. A single colony of bacteria was moved to 50 mL sterile LB medium and incubated at 37 °C with 60 rpm shaking for 18 h. Prior to inoculation, the bacteria concentration was adjusted to 2.5~10 × 10^5^ CFU/mL. 

#### 2.5.2. Antibacterial Activity Test

The determination of antibacterial activity against *E. coli* was conducted according to the ASTM E2149 standard with a slight modification. For each specimen, 0.1 g free UV film was immersed into a 20 mL LB culture medium with the addition 0.1 mL bacterial suspension. Two experiments were performed. In the static culture examination, the specimens were incubated at 25 °C for 24 h without shaking. In another dynamic culture examination, the specimens were incubated at 25 °C for 1, 3, and 5 h at 25 °C with 80 rpm shaking. 

#### 2.5.3. Determination of Antibacterial Activity

To quantify the bacterial strain in the culture flask, the spreading plate method was used. Ten-fold serial dilutions of bacterial suspensions were dispensed onto an LB agar plate, followed by incubation at 25 °C for 24–36 h. Colony counting was performed manually. For each assay, triplicate tests of each sample were conducted. Antibacterial activity was calculated by the following Equation (1).

Antibacterial activity (%) = (A − B)/A × 100(1)

where A and B are the average numbers of bacteria cells in the control and test group, respectively.

The optical density (OD) at 600 nm was detected by the ultraviolet-visible spectroscopy (DS5 Dual Beam, Edinburgh Instruments Ltd., Livingston, UK), and used for representing antibacterial activity. The OD was used to evaluate the growth of bacteria at 0, 1, 3, and 5 h for avoiding the errors resulting from the operating time delay. The calibration curve of ODs and bacterial concentrations (CFU/mL) in the antibacterial test were established to achieve the conversion of ODs and CFU/mL values.

### 2.6. Determination of the UV Film Properties

The attenuated total reflectance Fourier-transform infrared spectroscopy (ATR-FTIR) analysis of UV film was performed by a Perkin-Elmer spectrum 100 (Perkin Elmer, Shelton, CT, USA). The FE-SEM and EDS analyses were also used to observe and identify the surface and profile of films. The film pendulum hardness on glass sheets was measured by a König/Persoz pendulum hardness tester (Braive Instruments, Liège, Belgium) according to the ISO 1522 [28] with repeating numbers of 15. The film gloss was measured by a Dr. Lange 60^o^ reflectometer (Dr. Bruno Lange GmbH, Berlin, Germany), and 9 points in the specimen were recorded. The film abrasion resistance was conducted by a Taber abrasion tester (Model 503, Taber Industry, North Tonawanda, NY, USA). The average film loss mass after 1000 cycles with a CS-10 abrading wheel, 500 g loading and 5 repeating test was recorded. In the determination of film mass retention, free films were separated from the PET substrates and heated in a Soxhlet extractor (Dogger Co., New Taipei City, Taiwan) with refluxing acetone for 4 cycles (fill/siphon)/h. After 6 h extraction, the soaked films were oven-dried at 50 °C for 6 h and their mass retentions were calculated. The elastic modulus was measured by the TI 950 TriboIndenter^®^ (Bruker, Billerica, MA, USA) equipped with a diamond Berkovich probe. Dynamic mechanical analysis (DMA) was conducted by DMA8000 (PerkinElmer, Waltham, MA, USA) and the glass transition temperature (Tg) was determined by the Tan δ curve which was obtained by a single cantilever and standard aluminum sheet package method at a heating rate of 5 °C/min from 0 to 150 °C under the air atmosphere. The film lightfastness was measured by a Paint Coating Fade Meter (Suga Test Instruments, Tokyo, Japan). Films were irradiated by mercury light (H400-F, C Sun, New Taipei city, Taiwan) at a chamber temperature of 32 ± 5 °C for 100 h. The color change of film was measured by a spectrophotometer (CM-3600d, Minolta, Osaka, Japan). The lightness difference (ΔL*), the color difference (ΔE), and the yellow index difference (ΔYI) in the CIE L∗a∗b∗ system were used to evaluate the lightfastness of films. The thermogravimetric analysis (TGA) was conducted by a PerkinElmer STA 6000 (PerkinElmer, Waltham, MA, USA) with a heating rate of 10 °C/min^−1^ from 50 to 700 °C in the nitrogen atmosphere (San Yuan Gases Co., Ltd., Taichung, Taiwan).

## 3. Results

### 3.1. Manufacture and Identification of M(HEEP)_2_

**Figure 2:**
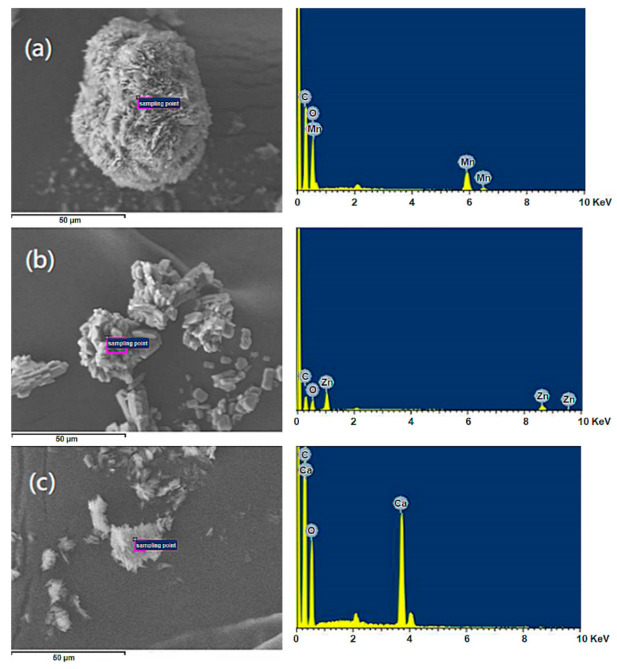
FE-SEM and EDS analyses of M(HEEP)_2_, M = (**a**) Mn (**b**) Zn (**c**) Ca.

In our previous research [26], the M(HEEP)_2_ with metals of Mn, Zn, and Ca were synthesized with yields of 43%–55%. In this study, a modified process by introducing a glass water separator and using thermal insulation materials to maintain the whole system temperature at 120 °C was investigated. In this modified synthesis, the condensed acetic acid and H_2_O, which were produced from the side reaction as shown in Figure 1, were removed from the reaction, and more M(HEEP)_2_ was produced. Furthermore, in the purified process of M(HEEP)_2_, the ethyl acetate/xylene eluents were used to replace the acetone/methanol/xylene used in our previous report [26]. The result showed that the low hygroscopicity of eluents could avoid the dissolution of M(HEEP)_2_ during the repeating purified processes. After the modified process, the yield of Mn(HEEP)_2_ could be increased from our previous study of 54% to 85%, Zn(HEEP)_2_ from 55% to 83%, and Ca(HEEP)_2_ from 43% to 73%. The lower yield of Ca(HEEP)_2_ was due to the washing consumption resulting in the low specific gravity of Ca(HEEP)_2_ powder and floating easily from the precipitation. The appearances of the M(HEEP)_2_ powders were milk-white in color, except the Mn(HEEP)_2_ powder was a pink color as the described in our previous report [26]. The morphology of M(HEEP)_2_ was inspected by the FE-SEM, and the results are shown in Figure 2. The Mn(HEEP)_2_ was piled to a spherical shape, a sheet shape of the Zn(HEEP)_2_, and a columnar shape of the Ca(HEEP)_2_. Compared with our previous study [26], the morphology and size of M(HEEP)_2_ powders were more homogeneous due to the reduced dissolving-reprecipitating behavior of M(HEEP)_2_ in the purification process, which led to more even nucleation produced [29]. In addition, the EDS analysis, as shown in Figure 2, also showed that the Mn(HEEP)_2_ contained 6.4% Mn element, 7.1% Zn element for the Zn(HEEP)_2_, and 6.5% Ca element for the Ca(HEEP)_2_. The results further revealed that the M(HEEP)_2_ powders were successfully and stably synthesized by the modified processes.

### 3.2. Antibacterial Activity of UV Films with Different Zn(HEEP)_2_ Additions

In our previous study [26], the waterborne urethane oil wood coating with 1 phr Zn(HEEP)_2_ addition had the best antibacterial activity against *E. coli* by an agar diffusion method. In this study, the Zn(HEEP)_2_ was also chosen to evaluate the antibacterial activity with the UV wood floor coating. The antibacterial activity of UV film with different Zn(HEEP)_2_ additions against *E. coli* by a static culture examination is displayed in Table 1. The colony counting was performed by the spreading plate method and optical density (OD) of LB culture medium, respectively, after 24 h incubation. In the blank group, the bacterial concentration was 2.70 × 10^8^ CFU/mL by spreading plate method, and it was similar to 2.55 × 10^8^ CFU/mL of immersing the raw UV film (0 phr). The result showed that the raw UV film had almost no antibacterial activity against *E. coli.* The bacterial concentrations of UV films with different Zn(HEEP)_2_ additions were 1.52 × 10^8^–1.58 × 10^8^ CFU/mL, and their antibacterial activity were 57–59%, meaning that the antibacterial activity could not be promoted with increasing Zn(HEEP)_2_ additions. 

For avoiding the errors resulting in the operating time delay, the OD was used to determine the bacterial concentration. Begot et al. [30] indicated that the OD has a higher correlation with a bacterial concentration in the low OD values. Consequently, the culture mediums after 24 h incubation were diluted to 25% *v/v*, and their OD values are listed in Table 1. The result showed that the OD value of blank was 0.400, which was similar to the 0.340 of the raw UV film. The Zn(HEEP)_2_-containing UV films had lower ODs of 0.234–0.244, indicating that the antibacterial activity of UV film with 4, 8, and 12 phr Zn(HEEP)_2_ had similar antibacterial efficiency. This phenomenon can be explained by two reasons. First, the logarithmic phase of *E. coli*’s growth is about 6–8 h [31,32] and it was reached in the stationary phase at the 8 h incubation in this study. Second, the UV films soaked in the culture medium and the Zn^2+^ released and the inhibitory efficacy against *E. coli* are highly dependent on the Zn^2+^ concentration [33,34,35]. However, the Zn^2+^-releasing behavior only occurred in the surrounding of UV film, and the growth of bacteria from a distance of UV film could not be inhibited.

**Figure 3:**
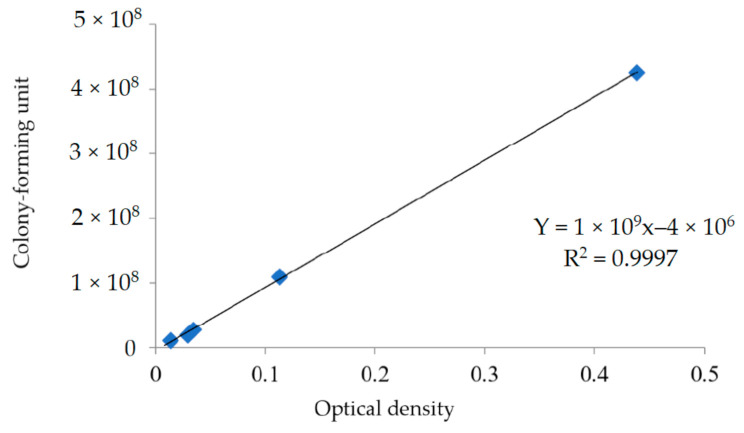
The calibration curve of OD_S_ and bacterial concentrations by the spreading plate method in the antibacterial test against *E. coli*.

For proofing the hypotheses, antibacterial experiments with a dynamic shaking culture of E. coli and a shorter incubation time were also examined. The UV film with 12 phr commercial nano-Ag was used as a comparison group. The calibration curve of ODs and bacterial concentrations by spreading plate method in the antibacterial test against *E. coli* was first established as shown in Figure 3, its correlation coefficient was 0.9997, and the results of bacterial concentration of UV films with different Zn(HEEP)_2_ additions are listed in Table 2.

The bacterial concentrations of all test conditions increased with increasing incubation time, while the Zn(HEEP)_2_-containing films had slightly higher bacterial concentration than the blank within 3 h incubation. This phenomenon may be caused by the rapid growth of *E. coli* during the logarithmic phase, and the data error generated from a few minutes of operating delay. 

After 5 h of experiment, the bacterial concentration of the blank and UV film without Zn(HEEP)_2_ were similar, 7.18 × 10^8^ and 7.21 × 10^8^ CFU/mL, respectively. The bacterial concentration of the 8 and 12 phr Zn(HEEP)_2_-containing UV films decreased from 7.21 CFU/mL of the 0 phr-containing one to 5.90 × 10^8^ and 5.18 × 10^8^ CFU/mL, respectively, indicating that the UV film with Zn(HEEP)_2_ addition possessed antibacterial efficiency. In addition, the 12 phr commercial nano-Ag-containing UV film also decreased to 6.84 × 10^8^ CFU/mL after 5 h of experiment. However, its antibacterial activity was lower than the UV film with 12 phr Zn(HEEP)_2_ addition. Sondi et al. [36] reported a similar result, i.e., that the nano-Ag with 100 μg/cm^3^ concentration cannot fully inhibit the *E. coli*’s growth with an initial bacterial concentration of 10^7^ CFU/mL. Lok et al. [37] indicated that the nano-Ag was oxidized by dissolved oxygen and transformed to Ag^2+^, and then the high antibacterial activity was generated. In this study, the nano-Ag may be blocked in the crosslinking structure of the resin in the UV film and separated with culture medium, meaning that less Ag^2+^ was generated and migrated to culture medium. Therefore, its antibacterial efficiency was inferior to the Zn(HEEP)_2_-containing film. Hsu et al. [14] prepared nanocomposites from a polyester-type waterborne polyurethane containing various concentrations of nano-Ag, and their results suggested that less ions were released from PU–Ag nanocomposites than in the direct addition of nano-Ag in the culture medium. In this study, in comparison with the nano-Ag, the Zn^2+^-generated limitation from the Zn(HEEP)_2_ was easy and a long alkyl chain of hydroxyethoxy ethyl group improved the penetration ability to inhibit the growth of attached *E. coli* [38,39]. 

**Figure 4:**
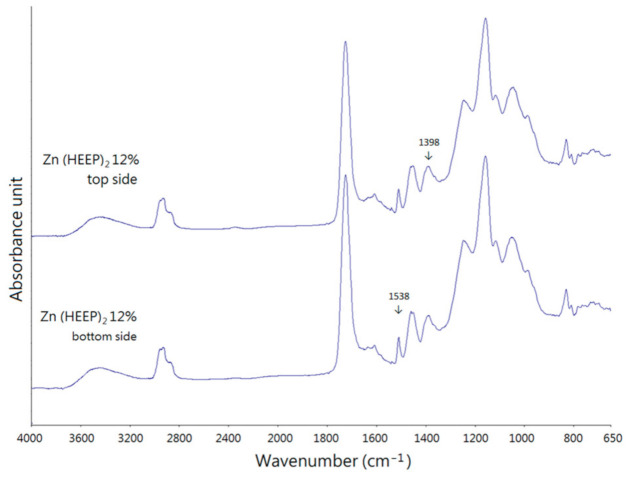
The ATR-FTIR analysis of the free UV film with 12 phr Zn(HEEP)_2_ addition: top side, and bottom side.

Furthermore, the metal-containing powders precipitate in the bottom of UV films during the curing process or that move to the surface through the eluents’ evaporation [40], which affect the antibacterial activity of the functional UV films and the top side and bottom side of free UV film, were determined by the ATR-FTIR to inspect the homogeneity of the Zn(HEEP)_2_ distribution, and the results are shown in Figure 4. The peaks of 1538 and 1398 cm^−1^ were associated with the symmetry and asymmetric stretch vibrations of COO^-^ structures of Zn(HEEP)_2_, and both of the two spectra were not noticeable different between the top and bottom surface of the UV film. This result demonstrated that the Zn(HEEP)_2_ were evenly filled in the UV film.

**Figure 5:**
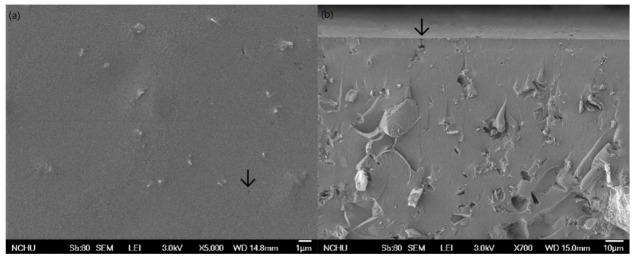
The FE-SEM inspection of UV film surface (**a**) and profile with 12-phr Zn(HEEP)_2_ addition (**b**).

For further proofing the Zn(HEEP)_2_ distribution in the films, the surface and profile of UV film were analyzed by the FE-SEM and EDS. The results of FE-SEM images are displayed in Figure 5. The Zn(HEEP)_2_ were filled in the UV film and a few pinholes were observed under the 5000× magnification, which had the size of 5–10 μm and were generated through the eluents evaporation.

**Figure 6:**
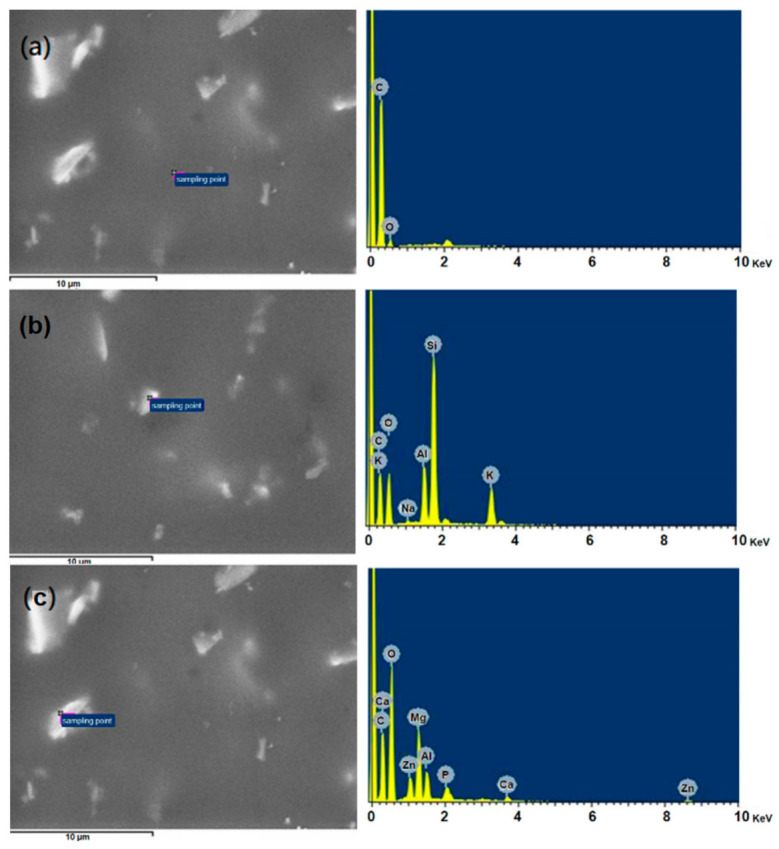
EDS mapping of UV films with different positions and Zn(HEEP)_2_ addition. (**a**) Specified smooth area of Zn(HEEP)_2_-containing UV film, (**b**) specified particle area of Zn(HEEP)_2_-containing UV film, (**c**) specified particle area of the raw UV film.

The EDS analysis of different positions of the UV films is shown in Figure 6. Figure 6a showed that the specified smooth area of Zn(HEEP)_2_-containing UV film was only carbon and oxygen elements, meaning that there was acrylate resin in the UV film. While the specific particle of raw UV film, as shown in Figure 6b, the Al, Si, and Mg elements were detected, and were the other additives in the UV coating. In the specific particle of UV film with 12-phr Zn(HEEP)_2_ addition, as shown in Figure 6c, the Zn element was detected. These results demonstrated that the Zn(HEEP)_2_ powders could be compatible with other additives and distributed in the UV film evenly. This phenomenon was also similar to the study results of Ramezanzadeh et al. [41], i.e., that when the ZnO nanoparticles (0–6.5 wt%) adding into the epoxy-polyamide coating, the nanoparticles tend to agglomerate and affect the bulk properties.

The effects of different Zn(HEEP)_2_ additions on the UV film properties are listed in Table 3. The results showed that film hardness increased slightly with increasing the Zn(HEEP)_2_, and the film with 12-phr Zn(HEEP)_2_ addition had the highest hardness of 103 sec when compared with the raw UV film (0 phr) of 97 sec. The Zn(HEEP)_2_ powders can be used as a body pigment to fill the void of the UV films and the hardness was improved. The film gloss decreased with increasing Zn(HEEP)_2_ addition, indicating that the Zn(HEEP)_2_ powders can also be used as a function of matting agent to reduce the gloss of UV film. The film abrasion resistance test showed that the UV film containing Zn(HEEP)_2_ had more mass loss from 13.4 (0 phr) to 17.1 mg (12 phr) after 1000 cycles of abrasion.

**Figure 7:**
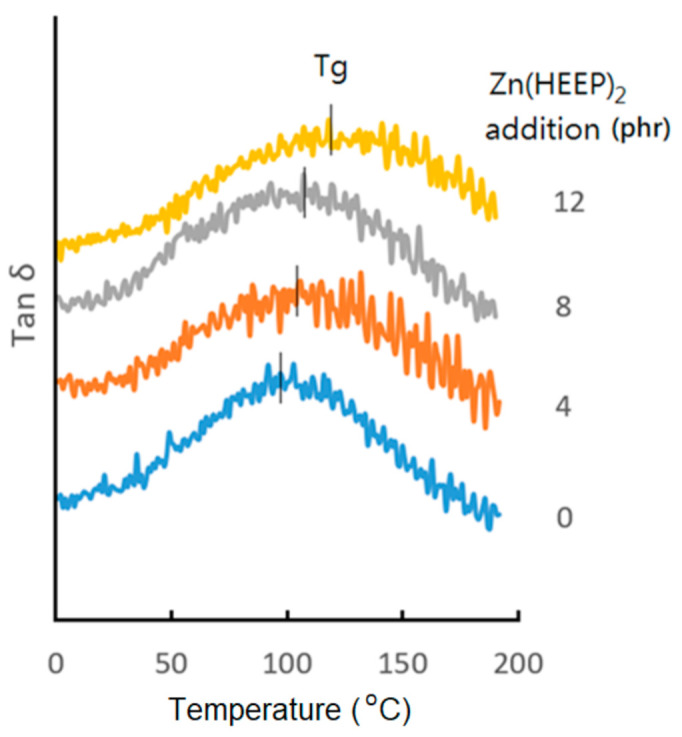
The Tan δ curves of UV films with different Zn(HEEP)_2_ additions.

The mass retentions of with and without Zn(HEEP)_2_–containing films were very similar, about 72.8–73.9%. The results indicated that the crosslinking structure of UV film was not affected by the addition of the Zn(HEEP)_2_ and the powder would be leached out in the experiment of siphon procedure. The modulus values in the indentation hardness test also revealed that the Zn(HEEP)_2_–containing UV films had higher modulus of 4.70–4.82 GPa than the raw UV film of 4.35 GPa. The Tan δ curves of UV films with different Zn(HEEP)_2_ addition are shown in Figure 7. The more Zn(HEEP)_2_ contained, the higher the Tg was. The 12 phr Zn(HEEP)_2_-containing UV film had the highest Tg of 120 °C when compared with the raw UV film of 96 °C. The results of hardness, modulus, and Tg values of the UV films indicated that the Zn(HEEP)_2_ powder has a function of antibacterial agent but also acts as a body pigment filler, which can reinforce the strength of the UV film under the critical pigment volume concentration (CPVC).

The lightfastness parameters of the UV film with different Zn(HEEP)_2_ additions after 100 h exposure to the UV light are listed in Table 4. The results showed that the lightness difference (ΔL*) of UV films were −0.3 to −0.7, indicating that the films were slightly darker after UV irradiation. However, the film containing 12-phr Zn(HEEP)_2_ had the lowest values of ΔL*, color difference (ΔE), and yellow index difference (ΔYI) of −0.3, 4.3, and 7.0, respectively, which were lower than those of raw UV films of −0.6, 5.3, and 8.5. The results revealed that the UV film with the most content of Zn(HEEP)_2_ in this study had the best lightfastness.

**Figure 8:**
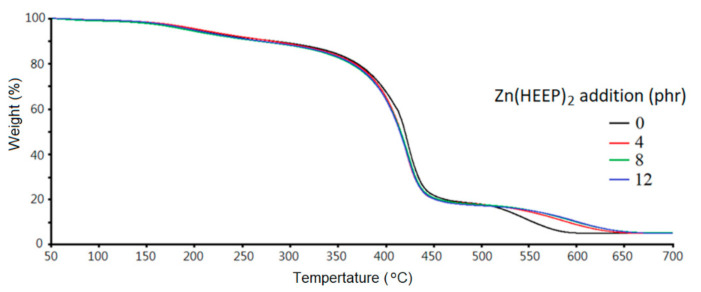
TGA curves of UV films with different Zn(HEEP)_2_ additions.

**Figure 9:**
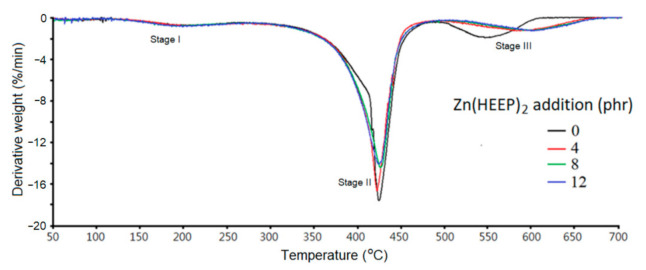
The DTG curves of UV films with different Zn(HEEP)_2_ additions.

The thermogravimetric (TG) analysis and derivative thermogravimetry (DTG) are shown in Figure 8 and Figure 9, respectively, and the thermal parameters are summarized in Table 5. The degradation of UV films consisted of three dominant stages in the pyrolysis process. In the first pyrolysis stage, the weight loss at temperature about 150–200 °C was due to the evaporation of moisture, trapped eluents, and low molecular weight of additives in the films, and all of the UV films with different Zn(HEEP)_2_ addition had the same behavior. 

The secondary pyrolysis stage at 350–480 °C corresponded to the degradation of the backbone of acrylate resin of the UV films and yielded various products resulting from the random main-chain scission, i.e., monomer, dimer, saturated diester, trimer, etc. [42]. In this stage, the UV film with Zn(HEEP)_2_ additions had lower pyrolysis onset temperatures of 391 to 393 °C and slower derivative weight loss of −14.0% to −16.7%/min at temperature of maximum decomposition rate (T_dmax_) than the raw UV film of 400 °C and −17.7%/min, respectively. 

In the third pyrolysis step, the previous produced char and products were further degraded to small molecular. The films with Zn(HEEP)_2_ addition had a noticeable increase of the onset of temperature (545–555 °C) and slower derivative weight loss of −1.3%/min at T_dmax_ than the raw UV film of 518 °C and −1.8%/min, respectively. These results showed that the UV film containing 12-phr Zn(HEEP)_2_ had the best thermal resistance. These results were also confirmed by Zafar et al. [5] and Hemalatha et al. [43], and described that adding inorganic powders can enhance the thermal stability of the polymeric films, and the adding of thermal-stable metal components in the films leads to a high residual weight after pyrolysis. However, the residual weight at 700 °C of UV films were 5.0–5.1% and had no obvious increase with increasing Zn(HEEP)_2_ additions in this study. This result could be explained by the low inorganic content, the Zn element of the Zn(HEEP)_2_ being only 7.1%. Therefore, the effect of the Zn(HEEP)_2_ on the residual weight of UV films could be ignored.

## 4. Conclusions

In the present work, the M(HEEP)_2_ powders with metals of Mn, Zn, and Ca were successfully and stably synthesized with yields of 73–85% through a modified process by introducing a glass water separator and using thermal insulation materials to maintain the temperature of the whole system at 120 °C. The raw UV film had no antibacterial activity, while the Zn(HEEP)_2_-containing UV films had antibacterial activity of 57–59%. In the antibacterial experimentations with dynamic shaking and a shorter incubation time, the UV film containing 12-phr Zn(HEEP)_2_ had a lower bacterial concentration of 5.18 × 10^8^ CFU/mL than the film containing commercial nano-Ag of 6.84 × 10^8^ CFU/mL, and which showed the best antibacterial activity in the different Zn(HEEP)_2_-containing films. The results of FTIR, FE-SEM, and EDS analyses demonstrated that the Zn(HEEP)_2_ powders were aggregated with the coating additives, evenly distributed, and packaged in the UV films. On the properties of UV film, the addition of Zn(HEEP)_2_ decreased the gloss and abrasion resistance and increased the hardness, Tg, thermal resistance, and lightfastness of UV films. However, they had no noticeable difference in the mass retention between the Zn(HEEP)_2_-containing UV films and raw UV film. For providing the antibacterial functional UV wood floor coating, the Zn(HEEP)_2_ can be used as a pigment and a matting agent, and the UV film containing 12-phr Zn(HEEP)_2_ was the most feasible and had the most potential among the different Zn(HEEP)_2_ additions in this study.

## Figures and Tables

**Table 1:** The antibacterial activity of UV films with different Zn(HEEP)_2_ additions against *E. coli* in the 24 h static culture test.

Zn(HEEP)_2_ Additions (phr)	Bacterial Concentration ^2^ (CFU/mL, × 10^8^)	OD Value ^3^
Blank ^1^	2.70	0.400
0	2.55	0.340
4	1.52	0.234
8	1.53	0.239
12	1.58	0.244

^1^ Without UV film; ^2^ Calculated by the spreading plate method; ^3^ The bacterial solution was diluted to 25% *v*/*v*.

**Table 2:** The antibacterial activity of UV films with different Zn(HEEP)_2_ additions against *E. coli* in the dynamic shaking culture test.

Zn(HEEP)_2_ Additions (phr)	Bacterial Concentration (CFU/mL, × 10^8^) with Incubation Time (h)			
	0	1	3	5
Blank ^1^	0.00	0.00	2.59	7.18
0	0.00	0.00	2.84	7.21
4	0.00	0.00	2.50	7.11
8	0.00	0.02	2.61	5.90
12	0.00	0.00	2.24	5.18
Ag (12 phr)	0.00	0.02	2.65	6.84

^1^ Without UV film.

**Table 3:** The film properties of UV films with different Zn(HEEP)_2_ additions.

Zn(HEEP)_2_ Additions (phr)	Hardness (König, sec)	Gloss (Gloss Units)	Abrasion Resistance (mg/1000 Cycles)	Mass Retention ^1^ (wt%)	Modulus ^2^ (GPa)	Tg ^3^ (°C)
0	97 ± 1	84.4 ± 0.7	13.4 ± 0.4	72.8 ± 0.4	4.35 ± 0.02	96
4	97 ± 1	83.5 ± 1.3	13.9 ± 0.5	73.8 ± 0.2	4.70 ± 0.05	104
8	98 ± 2	78.8 ± 2.9	14.5 ± 1.1	73.6 ± 0.9	4.82 ± 0.08	111
12	103 ± 2	73.1 ± 4.7	17.1 ± 1.3	73.9 ± 0.5	4.76 ± 0.04	120

^1^ Use acetone as a solution; ^2^ Determine by the indentation hardness; ^3^ Temperature of glass transition, determined by DMA.

**Table 4:** The lightfastness of UV film with different Zn(HEEP)_2_ additions.

Zn(HEEP)_2_ Additions (phr)	ΔL*	ΔE	ΔYI
0	−0.6	5.3	8.5
4	−0.7	5.8	9.3
8	−0.6	5.1	8.2
12	−0.3	4.3	7.0

**Table 5:** The pyrolysis parameters of UV films with different Zn(HEEP)_2_ additions.

Zn(HEEP)_2_ Additions (phr)	Stage II			Stage Ⅲ			Residual Weight at 700 °C (%)
	Onset (°C)	Derivative Weight Loss at T_dmax_^1^ (%/min)	T_dmax_ (°C)	Onset (°C)	Derivative Weight Loss at T_dmax_ (%/min)	T_dmax_ (°C)	
0	400	−17.7	422	518	−1.8	545	5.0
4	393	−16.7	421	545	−1.3	584	5.0
8	390	−14.4	424	551	−1.3	595	5.2
12	391	−14.0	423	555	−1.3	599	5.1

^1^ Temperature of maximum decomposition rate.

## Data Availability

Not applicable.
